# The Physiology of Secretion

**Published:** 1883-02

**Authors:** 


### The Physiology of Secretion.

Dr. Gamgee delivered an interesting address on the growth of our knowledge of the function of secretion before the biological section of the British Medical Association. The following are his general conclusions. The complicated studies of the physiology of secretion have brought into greater prominence the dignity, if I may use the expression, of the individual cell. The process of secretion appears as the result of the combined work of a large number of these units. Each, after the manner of an independent organism, uses oxygen, forms $CO^2$, evolves heat and derives its nutriment from the medium in which it lives, and performs chemical operations of which the results only are imperfectly known to us, and which depend upon peculiar endowments of the cell protaplasm, of which the causes are hidden from us. So long as the protoplasm is living, the gland cell retains its power of discharging its functions, and in many cases does so, so long as the intercellular liquid furnishes it with the materials required. In some cases, however, the gland cells are specially sensitive to a variation in the composition of the nutrient liquid, certain constituents of which appear to stimulate the protoplasm to increased activity. In the higher animals, the cells, particularly in certain glands, are in relation to nerves which, when stimulated, affect in a remarkable manner the transformations of their protoplasm, leading to an increased consumption of oxygen, an increased production of carbonic acid, an increased evolution of heat, and an increased production of those matters which the cell eliminates and which constitute its secretion.

This historical survey of the growth of our knowledge of the process of secretion exhibits the characteristic features of biological advancement. Comparative anatomy has been the foundation of observation of facts and physical experiment the road to physiological reseach. At various stages the value of hypotheses have been well illustrated, and whenever they have had to make way for the broader and truer interpretations suggested by the accumulation of facts and greater precision of observation, it has been demonstrated that the process of observation is not one of simple sight, but of complex ratiocination.—*Lancet, September* 2, 1882.

